# Effect of Buffer Layer Capacitance on the Electrical Characteristics of Ferroelectric Polymer Capacitors and Field Effect Transistors

**DOI:** 10.3390/ma14051276

**Published:** 2021-03-08

**Authors:** Eun-Kyung Noh, Amos Boampong, Yu Konno, Yuji Shibasaki, Jae-Hyun Lee, Yoonseuk Choi, Min-Hoi Kim

**Affiliations:** 1 Department of Creative Convergence Engineering, Hanbat National University, Yuseong-gu, Daejeon 34158, Korea; dmsrudsh@naver.com (E.-K.N.); jhyunlee@hanbat.ac.kr (J.-H.L.); 2 Department of Electronics Engineering, Hanbat National University, Yuseong-gu, Daejeon 34158, Korea; amosboampong.ab@gmail.com (A.B.); ychoi@hanbat.ac.kr (Y.C.); 3 Department of Chemistry & Biological Sciences, Faculty of Science & Engineering, Iwate University, 4-3-5 Ueda, Morioka 020-8550, Iwate, Japan; g0319065@iwate-u.ac.jp (Y.K.); yshibasa@iwate-u.ac.jp (Y.S.)

**Keywords:** ferroelectric, transistor, capacitor, buffer layer, capacitance, hysteresis, retention time

## Abstract

We demonstrated the effect of a buffer layer on the electrical characteristics of ferroelectric polymer capacitors and field-effect transistors. Various polymer materials with a dielectric constant between 2 and 42 were used to form buffer layers with a similar thicknesses, but with different capacitances. In order to evaluate the characteristics of the ferroelectrics with a buffer layer, the polarization–voltage characteristics of the capacitor, the transfer characteristics, and the retention characteristics of the transistors were investigated. As the capacitance of the buffer layer increased, high remnant polarization (*P*_r_), high hysteresis, and long retention times were observed. Exceptionally, when poly(methylmethacrylate) and rigid poly(aryl ether) (poly(9,9-bis(4-hydroxyphenyl)fluorene-*co*-decafluorobiphenyl)) were used as the buffer layer, *P*_r_ had a value close to 0 in the dynamic measurement polarization–voltage (P–V) characteristic, but the quasi-static measurement transfer characteristic and the static measurement retention characteristic showed relatively high hysteresis and long retention times. Our study provides a scientific and technical basis for the design of ferroelectric memory and neuromorphic devices.

## 1. Introduction

Ferroelectric polymer capacitors and field-effect transistors have attracted much attention due to their non-volatile memory characteristics and process compatibility for cost-effective fabrication [1,2]. Inorganic ferroelectrics such as BaTiO_3_ and HfO_2_ show high electrical performance, but require an expensive process due to the high-temperature vacuum thin-film deposition process [1,3]. On the other hand, polymer-based ferroelectrics are capable of low-cost, large-area processing through a solution process, and have advantages which are suitable for the fabrication of flexible electronic devices [1,2,4]. In particular, poly(vinylidenefluoride-trifluoroethylene) (P(VDF-TrFE)) exhibits ferroelectricity through a solution process at a low temperature, and thus it is most often used as an insulator of ferroelectric capacitors, and as a gate insulator of organic ferroelectric field effect transistors [5].

The copolymer P(VDF-TrFE) needs heat treatment in order to be formed into a β-phase with ferroelectric properties, in which the polymer chains move, resulting in crystallinity and high surface roughness [6]. In ferroelectric organic field-effect transistors (FeOFETs), the high surface roughness of the P(VDF-TrFE) decreases the crystallinity of the semiconductor film formed thereon, and thereby increases the trap density at the interface between the organic semiconductor and the insulator, which is a major obstacle for obtaining high electrical performance [1,7,8,9]. In order to reduce the high surface roughness of P(VDF-TrFE), a buffer/ferroelectric (B/F) bilayer in which a thin buffer layer formed on the ferroelectric layer was used. For this B/F bilayer structure, mixed studies have been reported on whether or not to maintain ferroelectricity in the B/F bilayer. Depending on the material used as the buffer layer, the mobility increases as the memory window is maintained [9,10,11], or the mobility increases as the hysteresis decreases [12,13]. D. Zhao et al. suggested that the capacitance of the buffer layer is the major condition for maintaining ferroelectricity in a capacitor with a B/F bilayer [14]. However, systematic studies on the ways in which the buffer layer affects the ferroelectricity of the B/F bilayer in the capacitor and transistor structures has not yet been sufficiently conducted.

In this work, we demonstrated the effect of the buffer layer on the ferroelectric characteristics of B/F bilayers in both capacitor and transistor structures. Various polymer materials with a dielectric constant (*ε_r_*) ranging between 2 to 42 were used to form buffer layers with a similar thickness, in order to achieve different capacitances. The polarization–voltage (P–V) characteristics in the capacitors, and the transfer and retention characteristics in the transistors were investigated. The large variations of these characteristics were monitored with various capacitances of the buffer layers, and it was deduced that, with the increasing the capacitance of the buffer layer, high remnant polarization (*P_r_*), high hysteresis (or memory window), and long retention times are obtained.

## 2. Materials and Methods

**Figure 1:**
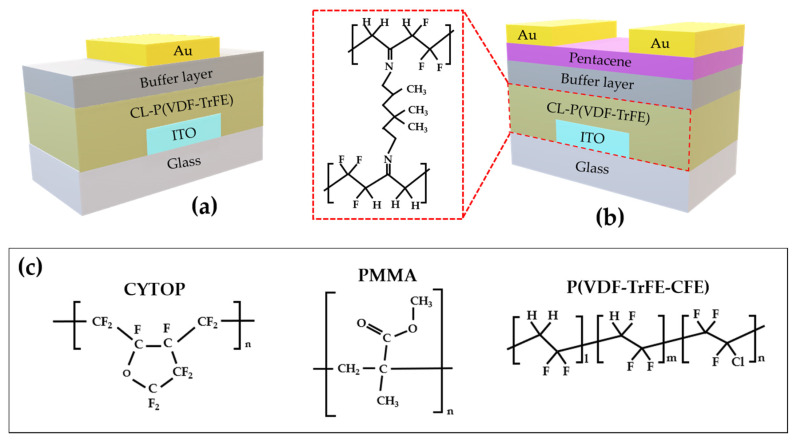
(**a**) Capacitor and (**b**) transistor structure with a B/F bilayer. (**c**) The chemical structure of the buffer layers: CYTOP, PMMA, and P(VDF-TrFE-CFE).

The main materials and fabrication methods of the ferroelectric polymer capacitors and field-effect transistors are described below, in Figure 1a,b, which shows the schematic images of a capacitor and an organic field-effect transistor with a B/F bilayer, respectively. The glass substrate with a 150 nm-thick ITO-bottom electrode for the capacitors (or the gate electrode of OFETs) was cleaned in an ultrasonicator for 15 min each using acetone and isopropylalcohol, and then using an ultra-violet treatment for 20 min. For the ferroelectric insulator, P(VDF-TrFE) and a cross-linker, 2,2,4(2,4,4)-trimethyl-1,6-hexanediamine (THDA), were dissolved in cyclopentanone separately in an 8 wt.% concentration. Both solutions were later mixed at a weight ratio of 95:5 and stirred for 3 h. The cross-linker reduces the effect of the upper buffer layer’s solvent on the underlying ferroelectric insulator. The P(VDF-TrFE):THDA solution was spin-coated onto the ITO-patterned glass substrates (3000 rpm, 30 s) and annealed for 2 h at 140 °C to increase the ferroelectricity and cross-linking in order to form a 520 nm-thick gate insulator. In order to form similar thickness of all of the buffer layers, each solution and solute were diluted or dissolved in different concentrations. CYTOP^TM^ CTL-809 M was diluted with CT-Solv.180 in a 2.25 wt.% concentration; rigid poly(aryl ether) (poly(9,9-bis(4-hydroxyphenyl)fluorene-*co*-decafluorobiphenyl)) (BHPF) and poly(methylmethacrylate) (PMMA) were separately dissolved in toluene at 0.9 wt.% and 1 wt.%, respectively; finally, poly(vinylidene fluoride-trifluoroethylene-chlorofluoroethylene) (P(VDF-TrFE-CFE)) was dissolved in cyclopentanone at 0.7 wt.%. All of the prepared buffer solutions were spin-coated onto the cross-linked P(VDF-TrFE) (CL-P(VDF-TrFE)) at a speed of 3000 rpm for 30 s and annealed for 60 min at 70 °C, except the CYTOP^TM^, which was annealed for 30 min at the same temperature. The thickness of the layers was measured using the α-step (Dektak-8 Surface Profiler) and summarized in Table 1.

A 50 nm-thick pentacene, a semiconductor layer, was thermally evaporated on the buffer layer at a rate of 0.5 Å/s. A 65 nm-thick Au was also thermally evaporated at a rate of 1.0 Å/s as a counter gate for the capacitor, and the source/drain (S/D) electrodes for the transistor as seen in Figure 1a,b, respectively. The source and drain electrodes were defined with a channel length and width of 200 μm and 1000 μm through a shadow mask. In order to measure the electrical characteristics of the FeOFETs, a semiconductor parameter analyzer (HP4155A, Hewlett Packard) was used. The capacitance measurement was performed using an impedance analyzer (CompactStat.h, Ivium technologies, Eindhoven, The Netherlands), and the polarization–voltage (P–V) characteristic was also measured using a ferroelectric analyzer (Precision LC Ⅱ, Radiant technologies. Inc., Albuquerque, New Mexico).

## 3. Results and Discussion

The effects of the B/F bilayer on the polarization–voltage (P–V) loops, transfer curves, and retention characteristics are described in this section. The ferroelectric properties of the B/F bilayers are further discussed as well. First, we calculated and derived the capacitance of the buffer layers. Because the actual buffer layer for the research is thin (about 70 nm), it is difficult to measure the capacitance due to a high gate current, and therefore the capacitance (*C***_i_**) and dielectric constant (*ε*_r_) were calculated from relatively thick layers.

The capacitance, thickness, and dielectric constant are presented in Table 1. The dielectric constants of CYTOP, BHPF, PMMA, and P(VDF-TrFE-CFE) are 1.90, 2.27, 3.94, and 42.24, respectively. In order to ascertain the actual capacitance of the buffer layers, relatively thin buffer layers were formed on the CL-P(VDF-TrFE), and their respective capacitances were calculated as $C_{i,\mathrm{buffer}}.$ Because buffer layers with similar thicknesses were used, as the dielectric constant increases, the $C_{i,\mathrm{buffer}}$ increases from 25.9 nF/cm^2^ (which is almost similar to CL-P(VDF-TrFE) capacitance) to 512.3 nF/cm^2^, which is almost 30 times higher than that of CL-P(VDF-TrFE).

**Figure 2:**
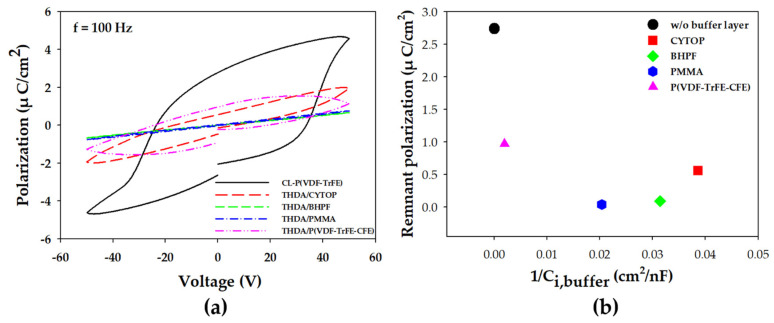
(**a**) Polarization–voltage characteristics of capacitors with B/F bilayers. (**b**) The remnant polarization (*P_r_*) monitored with *C*_i,buffer_. The thickness of the CL-P(VDF-TrFE) was used as the based reference (d = 0) to calculate the respective capacitance ($C_{i,\mathrm{buffer}}$) of the buffer layers.

In order to determine the effect of the capacitance of the buffer layers on the ferroelectric characteristics of the B/F bilayers, the P–V characteristics were measured as shown in Figure 2a. Based on the measured P–V characteristics, *P*_r_ was shown according to the change in capacitance of the buffer layers in Figure 2b. The *P*_r_ of CL-P(VDF-TrFE) is 2.77 μC/cm^2^, which is smaller than the conventionally-reported value of about 4.51 μC/cm^2^ of P(VDF-TrFE) [15]. This is because the P(VDF-TrFE) is cross-linked with THDA, which is a dielectric material, thereby increasing the ratio of the dielectric material and reducing the ferroelectricity [15]. When the capacitance of the buffer layer decreases, *P*_r_ sharply decreases. The *P*_r_ becomes almost zero when PMMA or BHPF is used. This decrease in *P*_r_ is because the surface charge of the buffer layer does not sufficiently compensate for the surface charge of the CL-P(VDF-TrFE), resulting in a depolarization field, which prevents the aligned ferroelectric dipole from being maintained [16]. In addition, because a part of the applied voltage is applied to the buffer layer, a voltage higher than the coercive voltage (*V*_coer_) is not sufficiently applied to the CL-P(VDF-TrFE), which is another reason why the ferroelectric dipole is not sufficiently aligned. Exceptionally, although the CYTOP buffer layer shows the smallest capacitance, a slight *P*_r_ of 0.56 μC/cm^2^ is obtained compared to BHPF and PMMA. This exceptional phenomenon may be a charge trap effect in the buffer layer with a low dielectric constant caused by the electric field from the ferroelectric dipoles. However, a more in-depth study is still needed.

**Figure 3:**
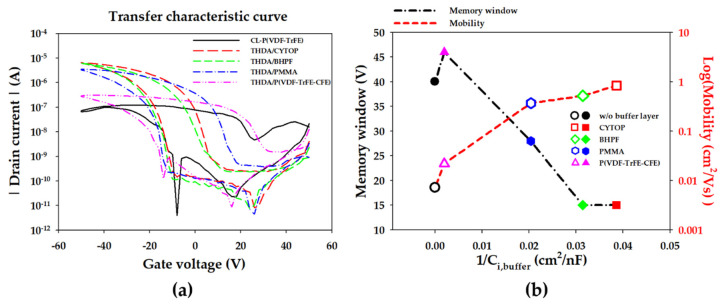
(**a**) Transfer curve of the FeOFETs with and without the buffer layers. (**b**) The memory window and mobility according to the capacitance of the buffer layer.

In order to ascertain the effect of buffer layers on the B/F bilayer in the transistor structure, the transfer characteristic curves of FeOFETs with buffer layers are shown in Figure 3a. All of the fabricated FeOFETs were measured by double sweeping gate voltage from 50 V to −50 V, at a drain voltage of −5 V; their calculated memory window (MW) and mobility, plotted against the capacitance of the buffer layers, are also shown in Figure 3b. The on-current (*I*_on_) of the FeOFETs with the buffer layer increases compared to the FeOFET without the buffer layer. This is attributed to the surface roughness of P(VDF-TrFE), with the root-mean-square roughness (*Z*_RMS_) of 2.8 nm decreasing due to the buffer layer [13], which reduces the trap between the organic semiconductor and the buffer layer [1]. The reduction of the surface roughness by the respective B/F bilayer also increases the interface capacitance, but the gate or leakage current is not significantly affected due to the relatively thick P(VDF-TrFE) gate insulators. The linear mobility increases as the dielectric constant of the buffer layer decreases; this is due to the interfacial polaronic effect on the charge carrier transport within the interface of the dielectric and the semiconductor [10]. The MW decreases as the capacitance of the buffer layer decreases, as shown in Figure 3b. Here, MW is defined as the difference between the gate voltages at which the average current of the logarithmic values of the on-current and off-current flows resulting from the dual *V*_coer_ from the forward and backward sweeps due to the ferroelectric polarizations switch. Compared to CL-P(VDF-TrFE), FeOFET without the buffer layer shows a high MW of about 40 V, and the MW of FeOFETs with a low capacitance buffer layer (BHPF and CYTOP) is narrowed to about 15 V. The decrease in MW is because, as shown in the P–V curves in Figure 2a, an insufficient voltage is applied to the ferroelectric P(VDF-TrFE) layer due to the voltage dividing effect within the buffer layer, and the uncompensated charges induce a depolarization field [14]. The transfer curve of the FeOFET with the P(VDF-TrFE-CFE) shows high hysteresis due to its high capacitance (512.3 nF/cm^2^), which is much greater than that of CL-P(VDF-TrFE), which has 18 nF/cm^2^. Interestingly, when the PMMA and BHPF buffer layers were used, the transfer curve of the FeOFET showed very clear hysteresis compared to the fact that there was almost no hysteresis in the P–V characteristic of the capacitor in Figure 2a. This was attributed to the P–V characteristic being a dynamic measurement observed with a 100 Hz triangle wave, whereas the transfer characteristic is a quasi-static measurement. As the time of the application of a voltage higher than the *V*_coer_ increases, the dipoles become aligned at relatively small voltages [17].

**Figure 4:**
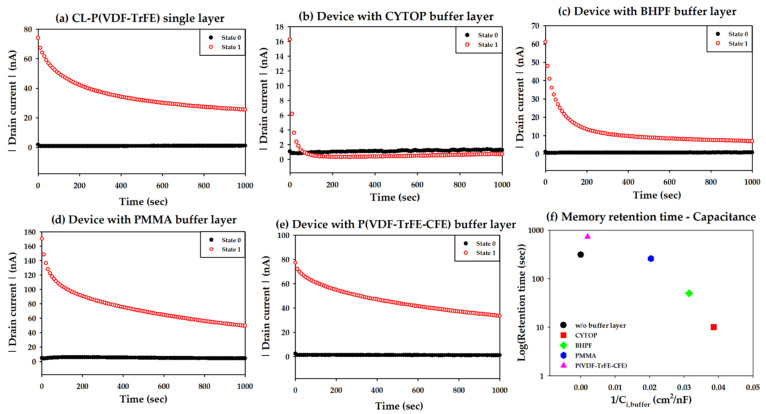
Memory retention characteristics of OFETs (**a**) without the buffer layer, and with a buffer layer of (**b**) CYTOP, (**c**) BHPF, (**d**) PMMA, or (**e**) P(VDF-TrFE-CFE). (**f**) Retention times corresponding to the capacitance of the buffer layers.

In order to investigate how long the dipole alignment of the ferroelectric is maintained, the retention characteristic of the OFETs was measured, as shown in Figure 4a–e. For the programing and erasing of the memory states, −50 V and +50 V were applied to the gate, respectively, and then a gate voltage of 0 V and a drain voltage of −5 V were applied for the reading operation [18]. In all of the FeOFETs, the initial memory off-current shows a small value compared to the memory on-current (*I*_memory,on_). The *I*_memory,on_ decreases over time, and the rate of the decrement depends on the capacitance of the buffer layer, which is summarized in Figure 4f. Here, the retention time was calculated as the time taken for the initial measured *I*_memory,on_ to be reduced by half.

Overall, as the capacitance decreases, the retention time decreases. This is because, similar to the P–V loops and transfer curves, the compensation charge of the buffer layer is insufficient, resulting in a depolarization field. When the PMMA and BHPF buffer layers were used, clear memory retention was shown, although the retention time was shorter than that of the FeOFET without the buffer layer and with the P(VDF-TrFE-CFE) buffer layer. Compared to the fact that there was almost no remnant polarization in the P–V characteristic of the capacitor with PMMA and BHPF buffer layers in Figure 2a, the retention time of more than several decades of seconds that was achieved is very interesting. The programming time in the retention measurement is much longer than the P–V dynamic measurement period, which means that the dipoles are able to be well aligned, hence the resultant retention time.

In general, when the capacitance of a buffer layer is high, it is expected to show high *P*_r_, high hysteresis, and long memory retention. Especially in the capacitor structure, it was reported that the higher the capacitance of the buffer layer, the higher the *P*_r_ [14]. Our results, as a whole, matched these characteristic tendencies well. In particular, the P(VDF-TrFE-CFE) with the high capacitance shows clear high *P*_r_, high hysteresis, and a long memory retention time. However, exceptional characteristics were observed due to the difference in the measurement speed of each P–V, transfer curve, and retention. As mentioned above, while the FeOFETs with the PMMA and BHPF buffer layer hardly show ferroelectricity in P–V, clear hysteresis in the transfer curves and relatively long retention times are exhibited.

## 4. Conclusions

The effect of the buffer layer on the electrical and memory characteristics of fabricated ferroelectric polymer capacitors and organic field-effect transistors was demonstrated by coating different buffer layers of variable dielectric constants on top of the cross-linked P(VDF-TrFE). The P–V characteristics of the capacitors, and the transfer and memory retention characteristics of the transistors were examined for the effect of various buffer layers on the ferroelectricity. The buffer layers (CYTOP, BHPF, PMMA and P(VDF-TrFE-CFE)) with varying capacitances and dielectric constants significantly showed trends in their hysteresis loops, memory window, mobility, ferroelectricity, and retention time. The higher the capacitance of the buffer layer, the higher the remnant polarization, the wider the hysteresis or memory window, and the longer the retention time. Our study provides a scientific and technical basis for the design of ferroelectric memory and neuromorphic devices.

## Figures and Tables

**Table 1:** Capacitance, thickness, and the dielectric constant for the ferroelectric and buffer layers.

	P(VDF-TrFE)	Cross-Linked P(VDF-TrFE)	CYTOP	BHPF	PMMA	P(VDF-TrFE-CFE)
*C*_i_ (nF/cm^2^)	16.1	18	2.00	0.501	2.05	44.0
*d* (nm)	510	520	920 *	4000 *	1700 *	850 *
*ε*_r_	9.3	10.3	1.90	2.27	3.94	42.24
*d*_buffer_ (nm)	-	-	72	63	71	73
*C*_i,buffer_ ** (nF/cm^2^)	-	-	25.9	31.8	49.1	512.3

* The layer is intentionally thickly formed in order to reduce the leakage current for the exact measurement. ** The values are calculated using $\;C_{i,\mathrm{buffer}}=C_{i}\times \frac{d}{d_{\mathrm{buffer}}}$.

## Data Availability

The data presented in this study are available within the article.
